# Imported cutaneous leishmaniasis: molecular investigation unveils *Leishmania major* in Bangladesh

**DOI:** 10.1186/s13071-019-3771-6

**Published:** 2019-11-07

**Authors:** Md Anik Ashfaq Khan, Rajashree Chowdhury, Rupen Nath, Sören Hansen, Progga Nath, Shomik Maruf, Ahmed Abd El Wahed, Dinesh Mondal

**Affiliations:** 1 Nutrition and Clinical Services Division, International Centre for Diarrhoeal Disease Research, Bangladesh, Dhaka, Bangladesh; 2 Division of Microbiology and Animal Hygiene, Georg-August-Universität Göttingen, Göttingen, Germany; 3 Surya Kanta Kala-azar Research Center, Mymensingh, Bangladesh

**Keywords:** Cutaneous leishmaniasis, Bangladesh, *Leishmania major*, Imported infection, Sequencing

## Abstract

**Background:**

The main clinical forms of leishmaniasis in Bangladesh are visceral leishmaniasis and post-kala-azar dermal leishmaniasis, which are caused by *Leishmania donovani*. Imported cutaneous leishmaniasis (CL) is emerging globally due mainly to increased human mobility. In recent years, several imported CL cases have also been reported in Bangladesh. Sporadic atypical cases of CL can be challenging for diagnosis and clinical management, while occurrence of infection on a frequent basis can be alarming. We report of a case of a Bangladeshi temporary-migrant worker who, upon return, presented development of skin lesions that are characteristic of CL.

**Methods:**

A serum sample was collected and tested with an rK39 immunochromatographic test. Nucleic acid from skin biopsy derived culture sample was extracted and screened with a real-time PCR assay which targets the conserved REPL repeat region of *L. donovani* complex. The internal transcribed spacer 2 region of the ribosomal RNA gene cluster was amplified and sequenced.

**Results:**

The suspect had a history of travel in both CL and VL endemic areas and had a positive rK39 test result. Based on clinical presentation, travel history and demonstration of the parasite in the skin biopsy, CL was diagnosed and the patient underwent a combination therapy with Miltefosine and liposomal amphotericin B. While typical endemic species were not detected, we identified *Leishmania major*, a species that, to our knowledge, has never been reported in Bangladesh.

**Conclusions:**

Proper monitoring and reporting of imported cases should be given careful consideration for both clinical and epidemiological reasons. Molecular tests should be performed in diagnosis to avoid dilemma, and identification of causative species should be prioritized.

## Background

Leishmaniasis is a group of insidious infectious diseases caused by species of the protozoan genus *Leishmania* transmitted through the bite of sand flies. It is classified into three clinical forms based upon the affected tissue, namely cutaneous (CL), mucocutaneous (MCL) and visceral (VL) leishmaniasis. The disease is endemic in many parts of the world. Bangladesh belongs to the endemic zones for VL as well as its skin complication known as post-kala-azar dermal leishmaniasis (PKDL), both of which are caused by *Leishmania donovani*. A regional initiative for VL elimination, known as the regional kala-azar Elimination Programme (KAEP) have contributed to a remarkable decline in the incidence rate of VL cases over recent years in Bangladesh and other endemic regions of the Indian subcontinent; it is now approaching the maintenance phase of elimination [1]. Patients affected by CL or MCL, on the other hand, are not usually found in Bangladesh, which might be due to the absence of specific transmitting vectors [2]. The diagnostic and clinical practices are well-defined in the local health care centres for VL and PKDL, which is not the case for CL or MCL. Upon occurrence, the atypical disease forms may cause diagnostic and clinical dilemmas with respect to clinical presentation, cross-reaction in serological tests, and treatment strategies [3, 4]. Systematic investigation of atypical cases and identification of the causative *Leishmania* species are also important for epidemiological reasons. Here, we report of a temporary-migrant worker who was diagnosed with CL after his return to Bangladesh. We identified *L. major* as the causative agent. To our knowledge, this is the first report of a *L. major* infected CL case in Bangladesh.

## Methods

### Case presentation

**Fig. 1:**
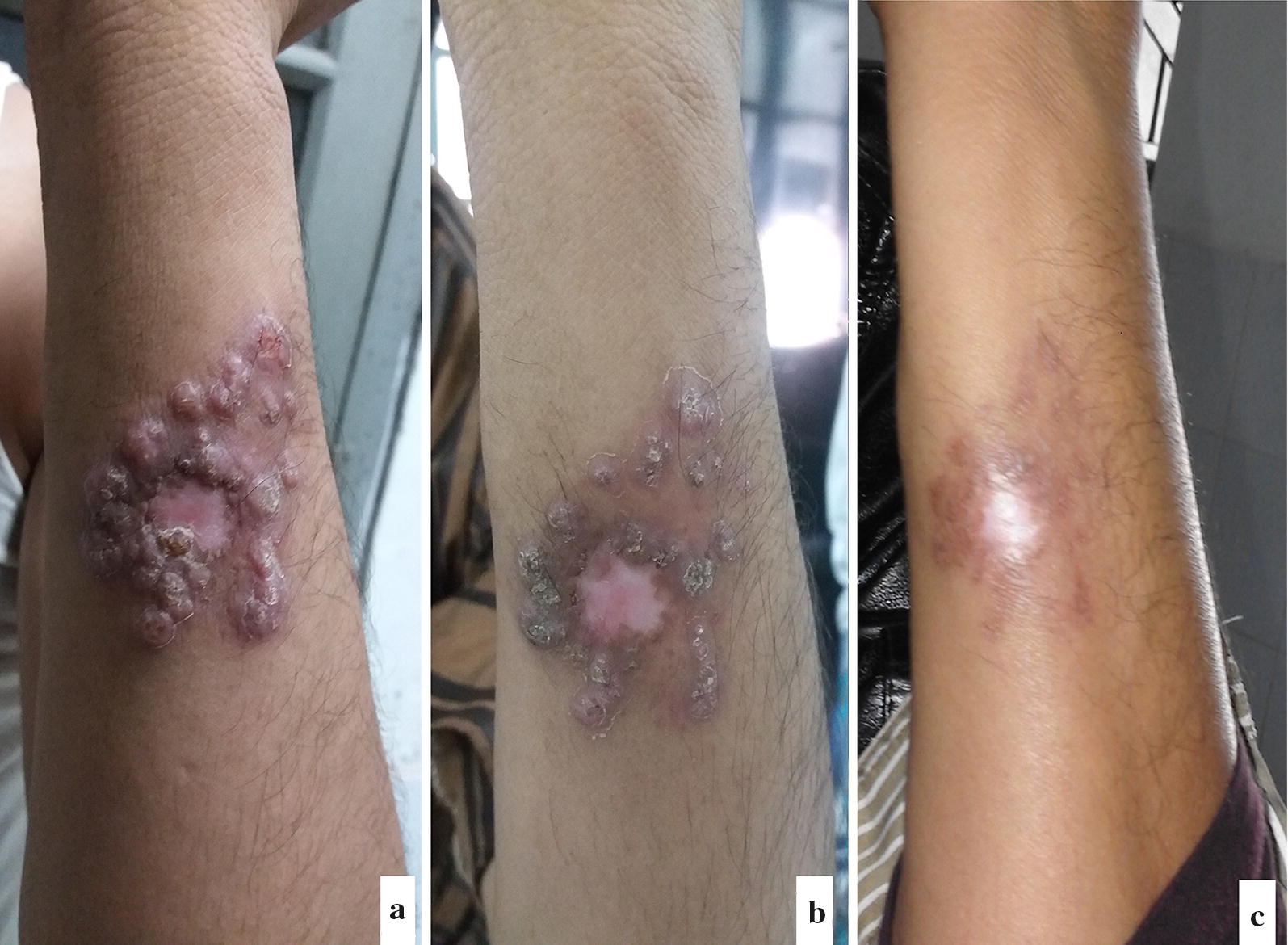
Ulcerative lesion surrounded by nodules on the lower left arm of the patient, October 2017 to January 2018, SKKRC hospital, Mymensingh before treatment (**a**), five days after treatment with 4 doses of AmBisome (**b**) and twelve weeks after treatment with Miltefosine (**c**)

A 40-year-old male was referred from the M.A.G Osmani Medical College Hospital, Sylhet to the Surya Kanta Kala-azar Research Center (SKKRC) hospital, Mymensingh in October 2017 as a suspected case of CL, with multiple skin lesions on his left forearm. However, no other anomaly such as fever, hepato-splenomegaly or mucosal lesion was observed. The patient had no history of VL, although he had been in to VL endemic areas of Bangladesh, and was found positive by an rK39 rapid immunochromatographic test (InBios International Inc., Seattle, Washington, USA). The period since the lesion first appeared was estimated to be three months, when he was working in the Kingdom of Saudi Arabia (KSA), a country known to be endemic for CL. Physical examination revealed one central depigmented ulcerated region surrounded by several hyperkeratotic, plaque like, sharply demarcated, painless papulonodular lesions (Fig. 1a) on his distal posteromedial aspect of the left forearm. Based on case history and clinical examination, a provisional diagnosis of CL was made and lesional biopsy was obtained for parasitological confirmation. Maintaining aseptic conditions, a ~ 3.0 mm in diameter skin snip was collected with a scalpel from the nodular lesions followed by direct microscopy of the Giemsa-stained thin biopsy smear, which revealed large macrophages containing abundant intracellular and extracellular amastigotes (3+ parasitemia grade: 1–10 parasites/microscopic field). One additional snip of a nodular lesion as well as pictures of the lesions were collected following the patient’s consent. The patient received a combination therapy of liposomal amphotericin B (AmBisome) at a dose of 20 mg/kg body-weight in four equally divided doses for four days. This was followed by an oral Miltefosine capsule for 12 weeks at a dose of 100 mg/day. The combination therapy resulted in a remarkable improvement, demonstrated by the dried up nodular crusts after five days (Fig. 1b), and disappearance of nodules leaving atrophic scars with hypopigmented spots in the middle after 12 weeks (Fig. 1c). No major side-effect was reported during the follow-up visits and the patient did not return with any relapse symptoms.

### Parasite culture and DNA extraction

The additional skin snip (~ 3.0 mm in diameter) collected from a nodular lesion was inoculated into RPMI-1640 culture medium with 10% FBS supplemented with penicillin-streptomycin. Two volumes each of stationary phase culture promastigotes were inactivated, stored in buffer AL (Qiagen) at a 1:1 ratio and sent to the Emerging Infections and Parasitology laboratory of International Center for Diarrheal Disease Research (Dhaka, Bangladesh). DNA was extracted by using a QIAmp Blood DNA Mini Kit (Qiagen).

### Real-time PCR and sequencing

A TaqMan probe based real-time (RT)-PCR assay, which targets the conserved region of *Leishmania* REPL repeats (L42486.1) of *L. donovani* complex, was carried out [5]. A threshold cycle (Cq) of 40- in a 45-cycle assay was considered positive. For species identification by sequencing, amplicons of the internal transcribed spacer region 2 (ITS2) were generated by PCR [6]. The amplicons were purified and prepared for Sanger sequencing by Microsynth Seqlab (Goettingen, Germany). Nucleotide BLAST search (NCBI) was used to estimate pairwise similarity of the tested sequence with reference *Leishmania* spp. genomes. A Tamura-Nei genetic distance model and neighbour-joining phylogenetic tree method for the derived ITS2 sequence was constructed together with sequences for *Leishmania* spp. with GENEIOUS v.9.1.6 (Biomatters Ltd., Auckland, New Zealand) using the incorporated tree builder at default settings.

## Results

**Fig. 2:**
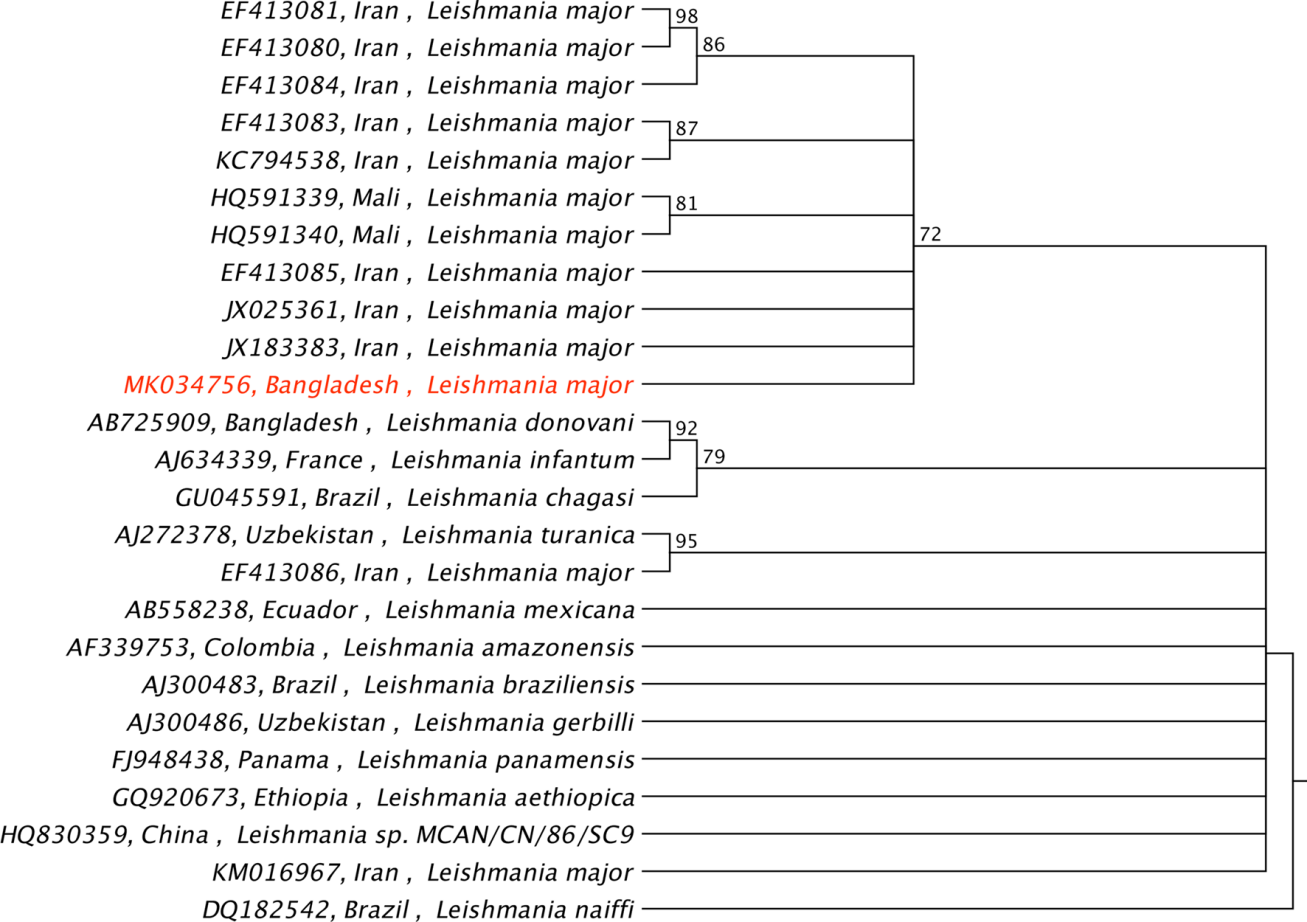
Phylogenetic relationships of *Leishmania* species showing the position of the present sample (red) of *Leishmania major*

The RT-PCR assay did not result in a positive detection of *L. donovani* DNA in the culture sample. For identification of *Leishmania* species, therefore, the PCR amplified 400-bp segment of ITS2 spacer was sequenced. The obtained sequence (Leish 17-832), which was assigned for species identification using nucleotide BLAST search (NCBI), showed pairwise similarity of 99% to *L. major* reference genome (GenBank: NC_007268) with a query cover of 100%. In contrast, for the closest reference genome strain of *L. infantum* (GenBank: NW_004057905.1), the pairwise identity and query cover was 88% and 90%, respectively (Table 1). The phylogenetic tree constructed for the obtained ITS2 sequence showed that *L. major*, originating possibly from Iran, shares a common ancestral node with the test sequence in a single branch (Fig. 2). The obtained nucleotide sequence was submitted to the GenBank database under the accession no. MK034756.

**Table 1:** Results of NCBI online nucleotide BLAST search of the newly generated sequence (Leish 17-832) using the NCBI Genomic Reference Sequences Database

Hit	GenBank ID	Pairwise identity (%)	Query cover (%)	e-value
*Leishmania major* strain Friedlin complete genome, chromosome 27	NC_007268.2	99	100	0.0
*Leishmania infantum* JPCM5 WGS CACT00000000 data, contig 37, whole genome shotgun sequence	NW_004057905.1	88	90	7e-105
*Leishmania donovani* BPK282A1 complete genome, chromosome 27	NC_018254.1	88	90	1e-102
*Leishmania mexicana* MHOM/GT/2001/U1103 WGS CADB00000000 data, contig 148, whole genome shotgun sequence	NW_003946471.1	87	87	7e-95

## Discussion

In Bangladesh, VL and PKDL are prevalent in endemic areas, whereas CL, a localized manifestation of nodular or popular lesion with ulceration, is not regarded endemic. Because the presentation of CL mimics common diseases like tuberculosis, anthrax and fungal infections [3], it may cause a diagnostic dilemma, especially in non-endemic regions, which can lead to inappropriate clinical management. In this report, national guidelines were followed to define the clinical CL suspect, and diagnosis was confirmed parasitologically [7]. Since the patient was rK39 ICT positive and had previously visited a VL endemic area, RT-PCR assay positivity would have suggested a chance of mixed infection [8] with *L. donovani*. On the other hand, although rK39 ICT is a specific antibody marker test for active VL detection, its cross-reactivity with sera from CL patients is also evident to some extent. Hence, the rK39 ICT test positivity could be associated either with an already healed infection with species that causes VL, species-specific cross-reactivity against CL-causing parasites [9–11], greater duration and severity of cutaneous infection [12] or region-specific phylogenetic proximity among species [13]. Finally, sequencing analysis of a species discriminatory segment of the ITS2 spacer [6] revealed that the obtained sequence (GenBank: MK034756) had almost 100% similarity with the reference genome sequence of *L. major* for absolute query cover (Table 1). The phylogenetic tree indicates that the test sequence shares common ancestry with *L. major* strains that originated in Iran (Fig. 2). This is consistent with case travel history, as *L. major* and *L. tropica* are the main dermotropic species in the CL endemic regions of countries of the Middle East including KSA. *Phlebotomus papatasi* (vector of *L. major*) and *P. sergenti* (vector of *L. tropica*) are the proven vectors of the parasite in this region [2]. In Bangladesh, however, *P. argentipes* is the only known vector of *L. donovani*. Although a possible variant of *L. donovani* has also been prevalently causing CL in neighbouring countries, India and Sri Lanka [14, 15], no such evidence is found in Bangladesh so far, and the other case reports of CL also indicated that the disease was imported from middle-eastern regions [16, 17]. Thus, CL can still be regarded as only an imported disease in Bangladesh.

Imported leishmaniasis has been becoming a globally emerging infectious disease in returned travellers; a 24-year analysis showed that more than 80% of those cases concerned CL [18]. Assessment of the risk of contracting CL, especially by Bangladeshi travellers, should be given careful consideration because the middle-eastern countries including KSA comprise one of the largest stocks of Bangladeshi migrants and temporary-migrant workers (> 3.0 million) [19]. Due to the self-healing nature of CL, many of them may go underreported upon return, and be asymptomatic or subclinical. Subsequently, the chances of genetic exchange between parasites can be relevant within the context, because *Leishmania* is capable of cross-species and intra-clonal mating, which may increase parasite fitness [20]. Moreover, *P. argentipes* is not competent to only *L. donovani*, but rather permissive to other pathogenic species including *L. major* [21]. Natural adaptation of a new *Leishmania* species to the endemic vector [22] or co-existence of species and/or genetic variants [15] in endemic zones are not unusual. More importantly, considering that co-endemic zones of VL and CL are emerging in neighbouring countries [15, 23], screening and examination of imported CL suspects will be crucial to estimate the occurrence rate, and address whether such atypical cases can potentially become a new challenge for the control initiatives against leishmaniasis in Bangladesh.

## Conclusions

In Bangladesh, imported cutaneous leishmaniasis is becoming more apparent. The imported CL case reported herein is, to our knowledge, the first evidence of *L. major*-derived pathology occurring in a Bangladeshi citizen. Our investigation indicates that presence of atypical cases in VL endemic areas can represent a diagnostic challenge, especially with antibody-based tests specific for active VL detection. Molecular tests should be performed in the diagnosis of such atypical cases to avoid dilemma. Furthermore, such cases should not be left out of epidemiological concern.

## Data Availability

Not applicable.

## Abbreviations

BLAST: basic local alignment search tool
CL: cutaneous leishmaniasis
ITS2: internal transcribed spacer 2
KAEP: kala-azar elimination program
KSA: Kingdom of Saudi Arabia
MCL: muco-cutaneous leishmaniasis
PKDL: post kala-azar dermal leishmaniasis
rk39 ICT: recombinant k39 immunochromatographic test
RT-PCR: real time polymerase chain reaction
VL: visceral leishmaniasis
